# OVX836 Heptameric Nucleoprotein Vaccine Generates Lung Tissue-Resident Memory CD8+ T-Cells for Cross-Protection Against Influenza

**DOI:** 10.3389/fimmu.2021.678483

**Published:** 2021-06-10

**Authors:** Judith Del Campo, Julien Bouley, Marion Chevandier, Carine Rousset, Marjorie Haller, Alice Indalecio, Delphine Guyon-Gellin, Alexandre Le Vert, Fergal Hill, Sophia Djebali, Yann Leverrier, Jacqueline Marvel, Béhazine Combadière, Florence Nicolas

**Affiliations:** ^1^ Research and Development Department, Osivax, Lyon, France; ^2^ Immunity and Cytotoxic Lymphocytes Team, Centre International de Recherche en Infectiologie, INSERM, U1111, Université Claude Bernard Lyon 1, CNRS, UMR5308, École Normale Supérieure de Lyon, Université de Lyon, Lyon, France; ^3^ Sorbonne Université, Inserm, Centre d’Immunologie et des Maladies Infectieuses (Cimi-Paris), Paris, France

**Keywords:** influenza vaccine, recombinant nucleoprotein, protection, cellular immunity, CD8+ T-cells

## Abstract

Tissue-resident memory (TRM) CD8+ T-cells play a crucial role in the protection against influenza infection but remain difficult to elicit using recombinant protein vaccines. OVX836 is a recombinant protein vaccine, obtained by the fusion of the DNA sequence of the influenza A nucleoprotein (NP) to the DNA sequence of the OVX313 heptamerization domain. We previously demonstrated that OVX836 provides broad-spectrum protection against influenza viruses. Here, we show that OVX836 intramuscular (IM) immunization induces higher numbers of NP-specific IFNγ-producing CD8+ T-cells in the lung, compared to mutant NP (NPm) and wild-type NP (NPwt), which form monomeric and trimeric structures, respectively. OVX836 induces cytotoxic CD8+ T-cells and high frequencies of lung TRM CD8+ T-cells, while inducing solid protection against lethal influenza virus challenges for at least 90 days. Adoptive transfer experiments demonstrated that protection against diverse influenza subtypes is mediated by NP-specific CD8+ T-cells isolated from the lung and spleen following OVX836 vaccination. OVX836 induces a high number of NP-specific lung CD8+ TRM-cells for long-term protection against influenza viruses.

## Introduction

Influenza A infection is a major cause of respiratory infections worldwide. Seasonal flu epidemics occur each year in autumn and winter, with a prevalence of 5 to 10%. They are caused by A/H1N1, A/H3N2, and B-type influenza viruses. Although most seasonal influenza infections are benign, they can cause hospitalization in severe cases, and even death in at-risk populations. Each year, up to 650,000 people die from influenza around the world (https://www.who.int/influenza/surveillance_monitoring/en/). The “at-risk” populations include elderly people, children, immunosuppressed individuals, and people with chronic diseases.

Influenza viruses are extremely variable. This variability is mainly due to the nature of RNA and the segmentation of the virus’ genome. Currently, the quadrivalent vaccines against seasonal influenza, which account for the majority of influenza vaccines worldwide, are inactivated, fragmented vaccines, administered in a single dose comprising 15 µg of hemagglutinin (HA) protein for four viral strains (1). These vaccines are composed of two type A (H1N1 and H3N2) viruses and two type B viruses, injected by the conventional IM route (1). The presence of antibodies directed against the HA glycoprotein on the virus surface which is subject to substantial immune selection pressure is considered the principal reference for protection against influenza viruses. However, vaccination strategies targeting influenza surface glycoproteins frequently have suboptimal effectiveness due to 1) virus mismatches (2, 3) and 2) low humoral responses in fragile populations (4). Nonetheless, CD8+ cytotoxic T-cells also play a role in the mechanisms that protect against influenza (5, 6). NP, in addition to the M1 and PB1 antigens, are significant sources of epitopes inducing cross-strain CD8^+^ T-cell responses (6–8). McMichael et al. performed a clinical challenge study showing that, in individuals lacking specific antibodies, high levels of CD8+ T-cells correlate with reduced viral shedding following experimental infection (5). During the 1980s, Doherty’s group also demonstrated the protective role of CD8^+^ T-cells against influenza (8). These influenza-specific T-cells play a crucial role in the control of influenza; they are capable of producing cytokines and killing infected cells (7, 9). Various authors have proposed that these cytotoxic CD8+ T lymphocytes might provide protection against multiple subtypes (i.e. H1N1, H5N1, and H3N2) (10). The persistence of cellular immunity against influenza virus variants may play an important role in reducing the severity of infections during epidemics and pandemics (11). This cellular immune memory against influenza viruses is conferred during infections (12). These CD8^+^ cell responses play a crucial role in viral infections, particularly in immunocompromised individuals (i.e. with an HIV infection or cancers) and the elderly (13, 14). Indeed T-cell responses may be a better correlate of protection in the elderly (5). In addition, their role as immune memory, able to persist and protect during influenza infections has moved forward substantially in the literature (15). Notably, the discovery of tissue localization of cellular immunity against infection has altered our understanding of adaptive immunity for protection. As a result, site-specific responses need to be taken into account in vaccine design (16, 17).

Lung tissue-resident memory (TRM) CD4^+^ and CD8^+^ T-cells generated following influenza infection have been shown to provoke viral clearance and survival after lethal challenge (18). Lung TRM T-cells are observed after viral infection and vaccination using live attenuated influenza viruses (LAIV) by the intranasal (IN) route (18). However, their induction remains insignificant when using recombinant protein or trivalent inactivated influenza vaccines. Compared with circulating T-cells, lung TRM cells protect animals against influenza infection (18). Mostly composed of CD8^+^ T-cells that recognize conserved epitopes, their induction *via* vaccination might be a key aim for effective heterosubtypic protection (6, 19).

OVX836 (18) is a recombinant protein vaccine candidate obtained by genetically fusing the NP sequence of the Influenza A/WSN/1933(H1N1) virus to the OVX313 sequence (oligomerization domain). By spontaneous oligomerization during the production process, OVX836 forms a stable homo-heptameric recombinant protein, comprising seven copies of the NP antigen (19). OVX836 demonstrated a protective efficacy in mice challenges using various influenza A subtypes, thus minimizing the risks of lower protection linked to antigenic drift and even mismatches (19). However, the mechanism of protection needs to be elucidated.

In the present study, we analyzed the mechanism of protection conferred by OVX836 and compared the immune responses and protection produced by three distinct NP proteins, all based on the NP sequence from the Influenza A/WSN/1933(H1N1) virus: monomeric E339A/R416A mutant NP (NPm), wild-type trimeric NP (NPwt), and heptameric NP (OVX836). Our findings demonstrate that the OVX836 vaccine, when compared to NPm and NPwt, generates higher proportions of lung TRM CD8^+^ T-cells with cytotoxic activity, producing a higher level of protection against influenza viruses.

## Methods

### Expression and Purification of Proteins

The amino acid sequence of NPm, NPwt, and OVX836 was based on influenza virus A/Wilson-Smith/1933. Synthetic genes, codon optimized for *Escherichia coli* expression, encoding NP-OVX313 (namely OVX836) and NPm (E339A/R416A) were purchased from ATUM Bio, USA. NP wild type (NPwt) was obtained by deletion of the OVX313 sequence from the OVX836 plasmid.

The recombinant NP proteins were produced using the *E. coli* BL21 (New England Biolabs) bacterial strain as previously described (19). After cell harvest by centrifugation, the pellets were resuspended in a phosphate buffer containing NaCl (supplemented with DNAse and RNAse for NPm), subsequently lysed by sonication on ice, and centrifuged. NPwt and OVX836 in supernatant were purified using a heparin affinity column followed by a diafiltration for OVX836 or gel filtration chromatography for NPwt. Supernatant containing soluble fraction of recombinant NPm was purified using a first ion exchange exclusion chromatography prior to the heparin and the gel filtration chromatography. Protein concentrations were determined by UV 280 nm measurement; their purity and identity were determined by SDS-PAGE, western blot and intact protein mass spectrometry.

### Mass Spectrometry

Measurements of the average mass of intact proteins were performed on a UHR-QqTOF mass spectrometer (Impact II, Bruker Daltonics) interfaced with a U3000 RSLC liquid chromatography system (CCSM, Lyon, France).

### Dynamic Light Scattering Analysis

The measurements were performed on a Malvern Zetasizer Ultra apparatus thermostatted at 25°C. The scattering intensity data, from three measurement angles (MADLS, multi-angle dynamic light scattering), were processed using the instrument software, transformed into the intensity and volume distribution to obtain the hydrodynamic diameter (D_H_) in each sample. The entire analysis was conducted in triplicate in 0.1 M Na/K_2_ phosphate, 0.5 M Na_2_SO_4_. The protein concentrations were 0.8 mg/ml (NPm), 0.4 mg/ml (NPwt), and 0.2 mg/ml (OVX836).

### Nano Differential Scanning Fluorimetry

nDSF (nano differential scanning fluorimetry) analysis (Tycho NT.6, Nanotemper) was performed to verify the structural integrity (or thermal stability) of NP constructs. The samples tested were the same as those used for the DLS experiments. After the capillaries were inserted into the Tycho NT.6, they were heated to 35–95°C at 20°C/min. The fluorescence was recorded during the thermal run, plotted as ratio and used to calculate the inflection temperature (Ti). These changes in fluorescence signal indicate transitions in the folding state of recombinant proteins. The Ti corresponds to the point at which half of the proteins in the solution have already unfolded.

### Electron Microscopy

Samples (concentrations around 0.002–0.02 mg/ml) were applied between a carbon and a mica layer. The carbon was then floated on the top of a 1% (w/v) sodium silicotungstate, pH 7.0 solution. The carbon film was covered with a copper grid. Both were fished out using a small piece of journal paper and air dried before insertion into the electron microscope. Charge-coupled device (CCD) frames were taken with a FEI T12 microscope operating at 120 kV and a nominal magnification of 30,000 times. The dilutions for EM were performed with the 0.01 M Na phosphate pH 7.3, 0.5 M NaCl buffer (NPm and NPwt) or water (OVX836) right before preparing the grid.

### Mice Immunizations and Influenza Virus Challenges

Six-week-old female C57BL/6 mice (Charles River Laboratories, Lyon, France) were used in all experiments. The animals were kept under specific pathogen-free conditions, with *ad libitum* access to food and water. All animal procedures were approved by the Institutional Animal Care ethics committee of the Plateau de Biologie Expérimental de la Souris (CECCAPP_ENS_2018_019, Lyon, France), and accreditations have been obtained from governmental agencies. The dose of 30 µg OVX836 used in this paper to characterize the mechanism of action of the vaccine was based on dose response studies in mice: this dose was selected as it provides high NP specific cellular responses and broad protection against influenza challenges. Mice were immunized twice, 21 days apart, with 30 µg of NPm and NPwt (0.536 µmol NP) and heptameric NP (OVX836, 0.476 µmol NP). Immunizations were performed by injection into the gastrocnemius muscle, with both injections being administered in the same hind limb. For immunogenicity studies, seven days after the second immunization, mice were sacrificed to collect serum, lungs, and splenocytes. All samples were processed individually immediately after collection.

For challenge studies, mice were infected 21 days after the last vaccination with the H1N1 influenza strain (A/California/07/2009) by intra-nasal administration of a 10^4.7^ Tissue Culture Infective Dose (TCID_50_) in 20 µl (10 µl/nostril) after ketamine/xylazine anesthesia. Weight was recorded for 10 days after the challenge. Animals that lost more than 20% body weight were euthanized according to institutional guidelines by cervical dislocation.

### Antibody Enzyme-Linked Immunosorbent Assay (ELISA)

Levels of immunoglobulin G (IgG) were measured in serum samples collected on D28 as previously described (19). Briefly, the 96-well ELISA plates were pre-coated with recombinant NPwt (OSIVAX) at 2.5 µg/ml overnight at 4°C. About 100 µl of serial 2-fold dilutions of serum (starting dilution 1/200) were added to each well and incubated for 2 h at 25°C. Bound antibodies were detected with goat anti-mouse IgG-HRP (Life Technology, USA) and finally, 100 μl of tetramethylbenzidine (TMB) (Interchim, France) substrate was added to each well. The antibody levels in the serum were expressed as a logarithm of endpoint dilution titer, defined as the reciprocal of the highest analytic dilution that gives a reading 3-fold over the mean O.D. 650 value of the negative-control mice serum at the 1/100 dilution.

### IFNγ ELISpot Assays

Influenza NP-specific T-cells secreting IFNγ were enumerated using an IFNγ ELISpot assay (Mabtech, Sweden). Lymphocytes were isolated from the spleen and the lung from individual mice as previously described (19). ELISpot plates were coated with the capture mAb (#3321-2H) then incubated overnight at 4°C according to the instruction manual of Mabtech. Then 2 × 10^5^ T-cells were cultured for 20 h at 37°C/5% CO_2_ with 2 µg/ml of recombinant NPwt or with 2 µg/ml of the NP_366–374_ (GenScript, Netherlands) immunodominant peptide epitope in C57BL/six mice. Concavalin A (Sigma-Aldrich, France) was used as a positive control and unstimulated splenocytes/lung cells were used as negative controls. Spots were counted with an ELISpot reader system (CTL-ImmunoSpot^®^ S6 Ultra-V, Germany). The number of protein- or peptide-reactive cells was represented as spot-forming cells (SFCs) per 2 × 10^5^ cells per well.

### Flow Cytometry Staining

Spleens and lungs were harvested at D28, after intravascular (IV) staining with 200 μl of anti-CD45-BV421 antibody diluted 1/300 in PBS 1×, for the identification of vascular T-cells (clone 30-F11; BD Biosciences, USA). Lungs and spleens were dissociated as previously described (19). Red blood cells from lungs and spleens were lysed and cells were counted with the EVE system (Witec AG, Swiss). 2 × 10^6^ cells of spleen and total lung cells were stained with 10 µl/sample of R-PE labelled Pro5 MHC H-2Db ASNENMETM (_366–374_) Pentamer (Proimmune, U.K.) for 20 min at room temperature, before viability staining with Fixable Viable Dye efluor 506 (eBioscience, USA). For lung tissue-resident T-cell analysis, cells were stained with a mix of antibodies: CD103-APC (clone 2E7, Biolegend, USA), CD62L-BV786 (clone MEL-14; Biolegend, USA), CD8-Super Bright 645 (clone 53-6.7, eBioscience, USA), CD3-APC-Cyanine 7 (clone 17A2, BD Biosciences, USA), CD4-FITC (clone GK1.5, BD Biosciences), CD44- PerCP-Cy5.5 (clone IM7, eBioscience, USA), and CD69-PerCP-Cy7 (clone H1.2F3; BD Biosciences). Flow cytometry was performed with a Fortessa™ and data were analyzed with Flowjo™ software (BD Biosciences, USA).

### DC Subset Isolation and *In Vitro* CD8+ T-Cell Stimulation

For isolation of dendritic cell (DC) subsets (CD11c^+^CD8^+^), a CD8+ Dendritic Cell Isolation Kit (Miltenyi, France) was used according to the manufacturer’s instructions. Briefly, splenocytes were incubated with a cocktail of biotin-conjugated antibodies (CD90, CD45R, CD49b), followed by anti-biotin microbeads to deplete T, B, and NK cells. The CD8α^+^DC subset was isolated with CD8^+^ selection. The CD8α+ DC subset was further purified using CD11c selection beads. The purity of the CD8α+ DC subset was 95%, as verified by flow cytometric analysis. The DC subsets were incubated with 2 µg/ml of either NPm, NPwt, or OVX836 at 37°C in the presence of 5% CO_2_ for 18 h. Cells were washed and expression of CD40 and CD86 activation markers was measured on CD8α^+^ DCs by flow cytometry. DCs were stained with a combination of Abs to murine CD11c-FITC (clone HL3, eBioscience, USA), B220-PE (clone RA3-6B2, eBioscience, USA), CD11b-APC-Cy7 (clone M1/70, eBioscience, USA), SiglecH-eFluor 450 (clone eBio440c, eBioscience, USA), CD86-PE-Cy7 (clone B7-2, BD Biosciences, USA), and CD40-APC (clone 3.23, eBioscience, USA). Then, antigen-loaded DCs were incubated *in vitro* with purified CD8^+^ T-cells from the spleen of mice immunized with 10 µg of NP_366–374_ peptide in IFA (Ratio 5:1). Naive DC-loaded with NP_366–374_ peptide and CpG 1826 (Invivogen, France), both at 2 µg/ml, were used as positive controls in the assay. After 2 h, brefeldin A (Sigma-Aldrich, France) was added at 5 µg/ml for 4 h of additional incubation. Lymphocytes were stained using CD8-Super Bright 645 (clone 53-6.7, eBioscience, USA) and CD3-APC-Cyanine 7 (clone 17A2, BD Biosciences, USA). For IFNγ intracellular cytokine staining, cells were fixed, permeabilized using CytoFix/CytoPerm (BD Biosciences, USA), and labeled with IFNγ−BV785 (clone XMG1.2, BD Biosciences, USA). Flow cytometry was performed with a Fortessa™ and data were analyzed with Flowjo™ software (BD Biosciences, USA).

### Cytotoxic Assay

Splenocytes (5 × 10^7^ cells/ml) from naive C57BL/6 mice were divided into two populations, labeled with two different concentrations of CFSE (Life Technology, USA). One population was pulsed with 4 μg/ml of NP_366–374_ peptide (GenScript, Netherlands) for 1 h at 37°C and treated for 15 min at 37°C with 0.5 μM CFSE (CFSE^low^). The other population remained un-pulsed and was treated with 5 μM CFSE (CFSE^high^). The CFSE^low^ (NP loaded) and CFSE^high^ cells (control) were mixed at a 1:1 ratio, washed twice in PBS + 2% FBS and incubated for 16 h with CD8+ T-cells obtained from the spleen or lung of animals immunized with OVX836, and purified by positive selection using CD8a (Ly-2) (Miltenyi Biotec, USA). This assay allows for the measurement of the intrinsic capacity of CD8+ T-cells to kill target cells to determine the actual value of cell specific lysis: 1) Ratio = %[CFSE^high^] peak/%[CFSE^low^] peak, 2) Percent Specific Lysis = [1 − (Control ratio/Experimental ratio)] × 100, as described previously (20).

### Adoptive Transfer Experiments and Influenza Virus Challenge

The experiment schema is represented in **Supplementary Figure 3**. Donor mice (n = 6–7 per group) were immunized twice (D0 and D21) intramuscularly (IM) with either 30 µg of OVX836 or Ovalbumin (OVA, 10 µg, Sigma-Aldrich, France) emulsified in IFA (Invivogen, France) or buffer. Mice of each group were sacrificed on D28 or D36. The lungs or spleens of the donor mice were processed individually to extract lung and spleen cells respectively as previously described (19). The cells were then pooled for cell sorting by positive selection with CD8α [MACS Isolation Kit CD8 (Ly-2)] or CD4 [MACS Isolation Kit CD4 (L3T4)] MicroBeads using MACS columns according to the manufacturer’s protocol (Miltenyi Biotech, France). The purity of CD8^+^ or CD4^+^ T-cells was >95% as determined by flow cytometry. Some 5 × 10^5^ lung-enriched CD8+ or CD4^+^ T-cells were transferred by the intravenous route to each recipient mouse 24 h before challenge. In addition, NP immune serum of each immunized mouse was collected at D36, pooled and each recipient mouse received 300 µl of this serum pool by the intraperitoneal route 24 h prior to influenza challenge. Six OVX836-vaccinated mice were used as positive controls in each challenge study. Recipient mice (n = 6) and positive control mice (n = 6) were then infected by intranasal administration of 10^4.7^ TCID_50_/20 µl (10 µl/nostril) of an H1N1 influenza strain (A/California/07/2009 or A/WSN/1933, Virpath, Lyon, France), after ketamine/xylazine anesthesia. Weight was recorded for 10 days after challenge. Animals that lost more than 20% body weight were euthanized according to institutional guidelines by cervical dislocation.

### Statistical Analyses

Statistical analyses and graphic representations were performed with Prism 8.0 (GraphPad Software Inc.). Statistical significance was determined using the unpaired, one-way analysis of variance (ANOVA) with Tukey’s multiple comparisons test or a non-parametric Kruskal–Wallis test followed by Dunn’s multiple comparisons test. Differences were considered significant if the p value was <0.05: *, <0.05; **, <0.01; ***, <0.001; ****, <0.0001. Survival rates of mice were compared using Kaplan–Meier survival analysis, and statistical significance was assessed using the Log-Rank (Mantel–Cox) test. The radar charts were designed with R (http://www.r-project.org/).

## Results

### NPm, NPwt, and OVX836 Proteins Display Different Physical Characteristics

**Figure 1:**
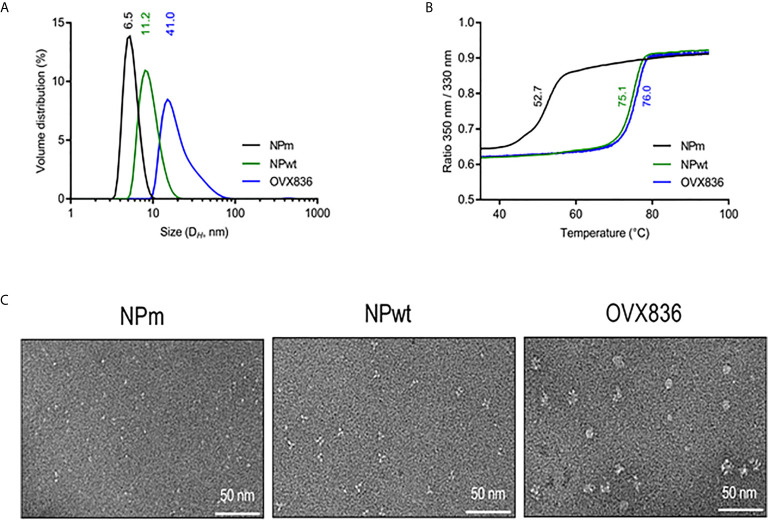
Characterization of influenza A nucleoprotein (NP) constructs. **(A)** Size distribution as measured by dynamic light scattering (left panel). The x-axis shows a distribution of size classes (nm) and the y-axis shows the relative volume distribution. Hydrodynamic diameters are obtained from scattering intensity DLS distributions. **(B)** Thermal stability as measured by nano differential scanning fluorimetry (right panel). The inflection temperature (Ti) values are mentioned. **(C)** Electron microscopy images. NP protein vaccines show different oligomeric states.

We have previously proposed that heptameric influenza A NP proteins can be obtained by fusing OVX313 to the C-terminal sequence of NP, conferring a higher level of immunogenicity to the NP protein (19). Here, we produced in *Escherichia coli* and purified the three following recombinant proteins: the E339A/R416A mutant of strain A of NP (NPm), wild-type NP (NPwt), and NP-OVX313 (OVX836), prior to studying their immunogenicity. **Table 1** summarizes the structure and physicochemical characteristics of the three NP proteins. First, intact protein mass spectrometry confirmed the homogeneity and the expected mass of NPm and NPwt. Under conditions leading to complete reduction of the disulfide bonds, the primary structure of the OVX836 subunit was also ascertained by high-resolution mass spectrometry. Then, we assessed the degree of oligomerization of these three NP proteins by measuring their hydrodynamic diameter using dynamic light scattering. As shown in **Figure 1A** and in **Table 1**, we found that NPm remains monomeric in solution (D_H_ 6.5 nm), confirming previous findings (21, 22). NPwt protein forms oligomers with a D_H_ of 11.2 nm, whereas OVX836 displayed larger oligomers with a D_H_ of 41.0 nm and a rather multimodal size distribution. As shown in **Figure 1B** and in **Table 1**, the unfolding profiles of the three proteins were compared by nano differential scanning fluorimetry (nanoDSF). NPwt and OVX836 thermally unfolded at 75–76°C, a relatively high temperature, as compared to that of NPm (52–53°C). NPm is thus intrinsically more thermolabile than its wild-type counterparts. By negative stain electron microscopy (EM), we confirmed that NPm was monomeric (**Figure 1C**, left image) and showed that NPwt forms mainly trimers in solution (**Figure 1C**, middle image). When analyzing OVX836, EM images showed mainly heptameric NP structures, as well as higher-order structures formed through protein/protein interactions (**Figure 1C**, right image).

**Table 1:** Structural characteristics of the NP proteins.

Construct	Mass (Da)^+^	D_H_ (nm)^*^	EM^**^	Length (nm)^**^	Ti (°C)^++^
NPm	56103.77	6.5 ± 0.3	Monomer	n/d	52.7
NPwt	56246.89	11.2 ± 0.3	Trimer	13 ± 2	75.1
			Heptamer to oligo-heptamer	18 ± 2 (heptamer)	
OVX836	62710.56	41.0 ± 1.4		43 ± 16 (di-heptamer)	76.0

The observed average masses of recombinant proteins by mass spectrometry agree with theoretical primary structures (^+^). Hydrodynamic diameters (D_H_) obtained from scattering intensity DLS distributions (*). Main species and lengths seen in electron micrographs (**). Inflection temperatures (Ti) obtained from melting curves by nDSF measurements (^++^).

In conclusion, we characterized three NP proteins as defined by their physicochemical characteristics: monomeric NP (NPm), trimeric NP (NPwt), and heptameric NP (OVX836 vaccine candidate). Then, their immunogenicity and protective efficacy were compared in mice.

### Compared to NPm and NPwt, OVX836 Vaccine Induces Higher Numbers of Persistent NP-Specific CD8+ T-Cells in Lung Tissue and Spleen, Providing Better Protection Against Influenza

**Figure 2:**
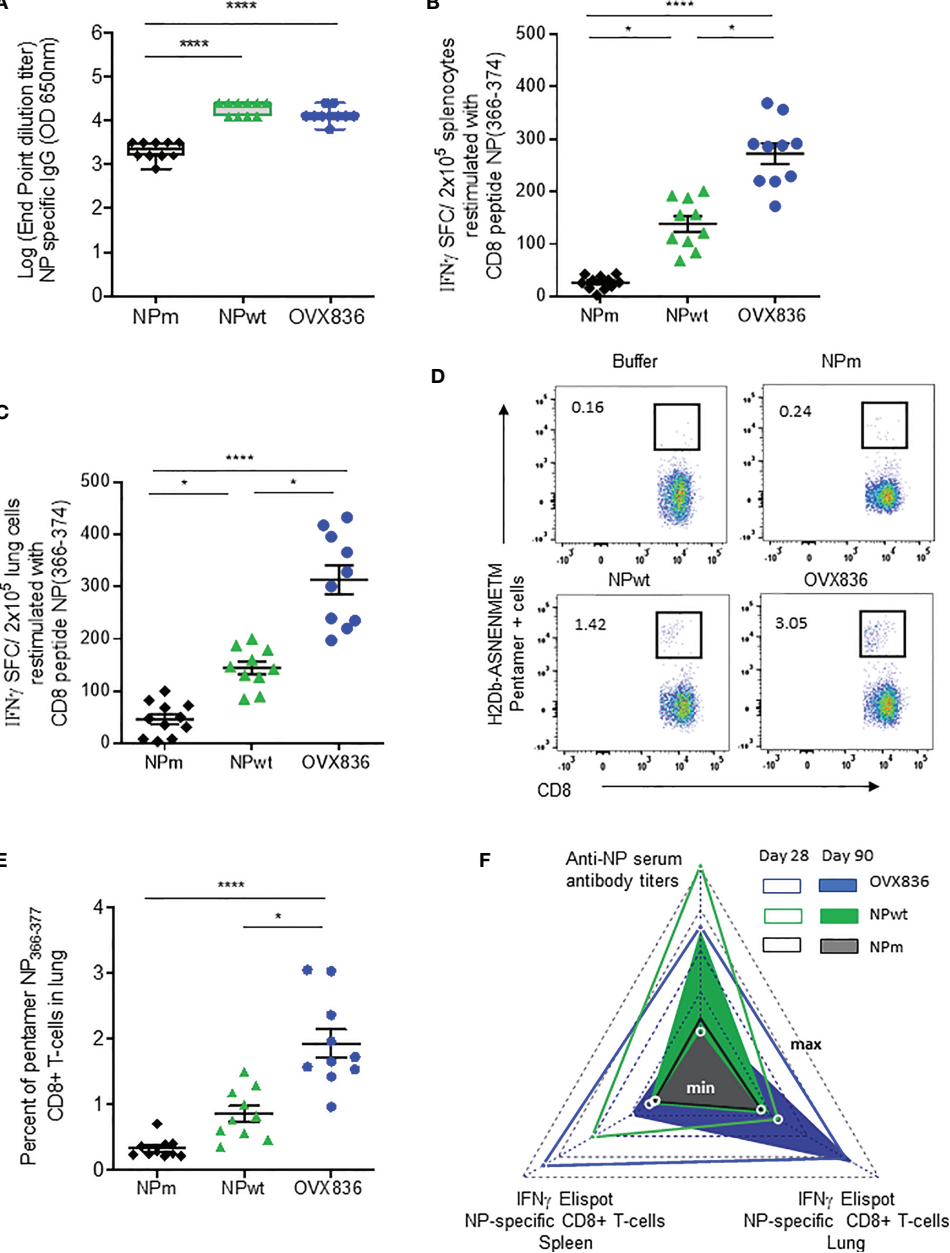
OVX836 vaccine induces higher numbers of persistent NP-specific CD8+ T-cells in lung tissue and spleen compared to NPm and NPwt. C57BL/6 female mice (n = 10) were immunized twice (D0, D21) with 30 µg of NPm, NPwt, and OVX836 by the IM route. **(A)** NP-specific IgG were measured by ELISA in serum at D28. Levels of IgG are expressed as Log (endpoint dilution titer) and represented in box-and-whisker plots. **(B, C)** NP_366–374_-specific IFNγ secreting T-cells (spot-forming cells (SFC)/2 × 10^5^ cells) were measured by ELISpot in the spleen **(B)** and in the lung **(C)** at D28. **(D, E)** In the lung tissue, NP_366–374_-specific CD8+ T-cells were detected by flow cytometry using pentamer staining (H2Db-NP_366–374_). Representative flow cytometry plots show frequencies of NP_366–374_-specific CD3+CD8+ T-cells in the lung **(D)**. Percentage of NP_366–374_-specific CD3+CD8+ T-cells in the lungs of mice **(E)**. Individual data, mean (line), and SEM are represented, n = 10 mice per group in two independent experiments. Differences were assessed by one-way ANOVA followed by Tukey’s multiple comparison test or with a Kruskal–Wallis test followed by Dunn’s multiple comparison test. *p < 0.05; **** for p < 0.0001. **(F)** Radar chart presents the minimum (min) and maximum (max) values for each assay as indicated in log10 scale. Comparing the mean of antigen-specific immune responses at D28 (empty triangle) and D90 (plain triangle): NPm (black), NPwt (green), and OVX836 (blue).

In order to measure the immunogenicity and protective efficacy of NPm, NPwt, and OVX836, we immunized mice twice (D0, D21) by the IM route. Humoral and cellular immune responses were measured at D28 by ELISA, IFNγ ELISpot, and H-2Db NP_366–374_ Pentamer staining (**Figure 2**). Whereas anti-NP IgG levels were significantly higher after NPwt and OVX836 compared to NPm immunization (p <0.0001) (**Figure 2A**), NP_366–374_-specific CD8^+^ T-cell responses were significantly higher after OVX836 compared to NPwt (p <0.05) and NPm (p <0.0001) immunization, as measured by IFNγ ELISpot assays in both the spleen (**Figure 2B**) and lung (**Figure 2C**). The superior ability of OVX836 over NPwt and NPm to generate lung tissue-associated CD8 cellular responses was confirmed by H-2Db NP_366–374_ Pentamer staining of CD8^+^ T-cells (**Figures 2D, E**).

We also measured cellular and humoral effector memory responses at D90 (**Supplementary Figure S1** and **Figure 2E**). Whereas humoral responses were maintained at D90 for all NP proteins (**Supplementary Figure S1A**), only OVX836 induced persisting NP_366–374_-specific IFNγ-producing CD8+ T-cells in the lung and spleen at this late time point (**Supplementary Figures S1B–E**). All immunogenicity results are compared and visualized in a radar chart (**Figure 2F**). Interestingly, at the memory phase, OVX836 induced persistent NP_366–374_-specific IFNγ-producing CD8^+^ T-cells, especially in the lung, whereas NPwt vaccination promoted persistent anti-NP antibodies (Abs), however, with reduced cellular responses over time. NPm is overall less immunogenic when compared to NPwt and OVX836.

**Figure 3:**
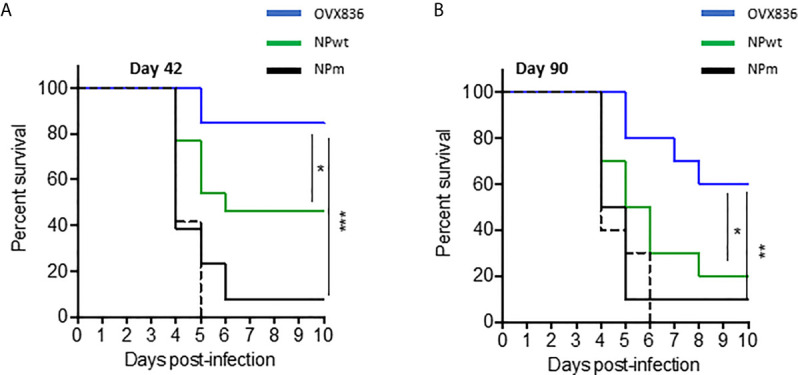
Long-term protection against viral challenges following OVX836 vaccination compared to NPm and NPwt. C57BL/6 mice (n = 12) were immunized twice 3 weeks apart (D0, D21), with 30 µg of NPm, NPwt, OVX836, or buffer (control mice) by the IM route. **(A)** 42 days and **(B)** 90 days post-first vaccination, mice were infected by IN route with 10^4.7^ TCID_50_/20 μl (10 μl/nostril) of influenza H1N1 A/California/7/2009. Graph shows the percentage of survival observed among each group of mice. Buffer (dashed black), NPm (plain black), NPwt (green), and OVX836 (blue). *p < 0.05, **p < 0.01 and ***p < 0.001 by Log-Rank (Mantel–Cox) test.

Lethal challenge studies using the A/California/07/2009 virus were performed in mice vaccinated with OVX836, NPwt, and NPm both at D42 (**Figure 3A**) and D90 post-first vaccination (**Figure 3B**). A significantly higher protection rate was observed after OVX836 vaccination at the effector phase (D42) compared to NPwt (p <0.05) and NPm (p <0.001). At the memory phase (D90), the protection against lethal influenza challenge was lost following vaccination with NPwt and NPm, whereas it was maintained, although slightly decreased, after vaccination with OVX836 (**Figure 3B**). The level of protection was associated with a high number of NP-specific CD8^+^ T-cells as observed in OVX836 compared to NPm and NPwt-immunized mice. In addition, the pronounced persistence of NP-specific CD8+ T-cells responses in the lung **(** **Figure 2F** and **Supplementary Figure S1**) suggests the important role of the lung CD8+ T-cells in animal protection.

**Figure 4:**
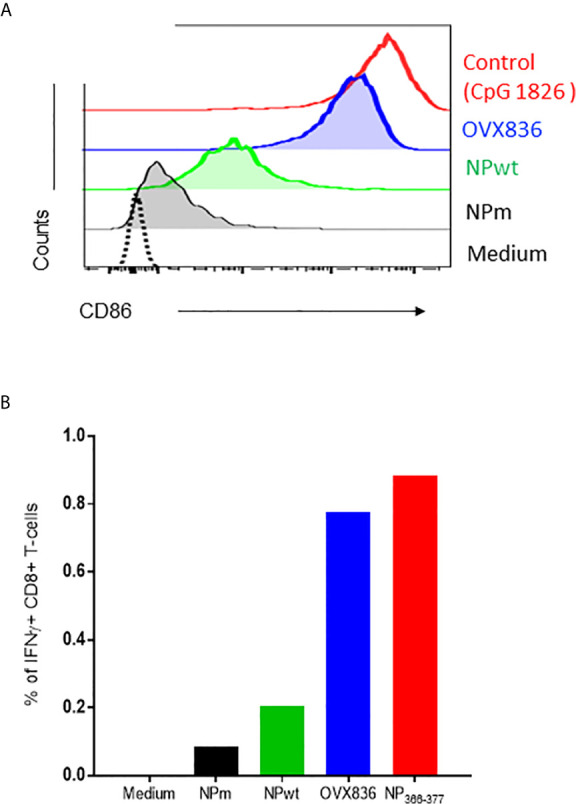
OVX836 activates CD8α+ DC and promotes effector T-cell responses *in vitro.* **(A)** Mean CD86 expression on purified CD8a^+^ DCs incubated for 18 h with 2 µg/ml NPm (36 nM NP), NPwt (36 nM NP), OVX836 (32 nM NP), or a positive control. **(B)** Graph shows the percentage of specific CD8+ T cells that were positive for IFNγ, measurement of IFNγ production by NP-specific CD8^+^ T-cells incubated for 6 h with antigen-loaded DCs (as indicated) at a 5:1 (DC/CD8) ratio, then analyzed by flow cytometry. CD8^+^ T-cells used in the assay were isolated from the spleen of mice immunized with 30 µg of NP_366–374_ peptide in IFA. NP_366–374_ was used as a positive control in the assay. Results are representative of two independent experiments.

In order to understand the mechanism of induction of CD8+ T-cell responses, we hypothesized that OVX836 might better stimulate these cells by inducing activation of DC. To test this hypothesis, we performed *in vitro* experiments assessing antigen presentation to CD8+ T-cells. First, we observed that purified CD8α^+^CD11c^+^ DC expressed a higher level of CD40 (data not shown) and CD86 surface activation markers when incubated with OVX836 compared to NPm and NPwt (**Figure 4A**). Then, antigen-loaded CD8α^+^CD11c^+^ DC were incubated with NP_366–374_-specific CD8^+^ T-cells isolated from mice immunized with NP_366–374_ peptide plus Incomplete Freund Adjuvant (IFA). We observed a higher production of IFNγ by NP_366–374_-specific CD8^+^ T-cells when using DC incubated with NP_366–374_ (positive control) and OVX836 compared to NPm and NPwt-loaded DC (**Figure 4B**). These results show that the OVX836 vaccine promotes DC activation favoring higher CD8^+^ T-cell effector responses compared to NPm and NPwt.

### OVX836 Generates a Higher Number of Lung TRM CD8^+^ T-Cells With Cytotoxic Activity

**Figure 5:**
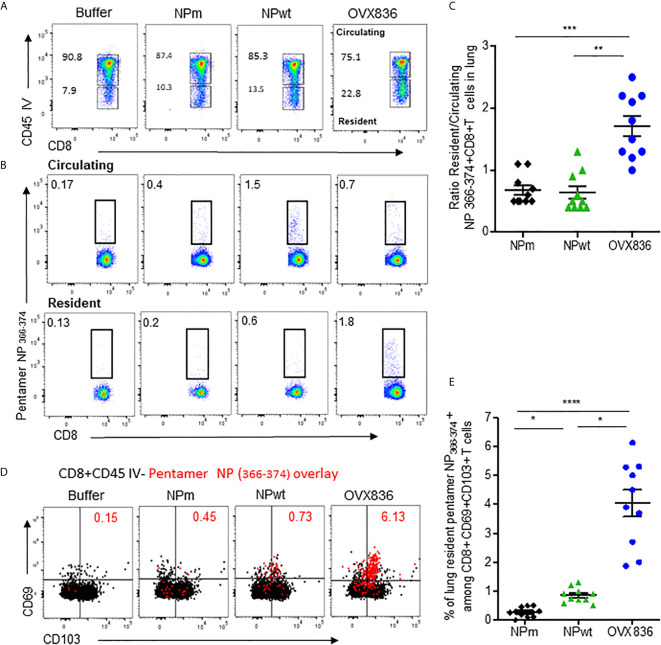
OVX836 vaccine generates lung NP-specific CD8+ TRM cells. C57BL/6 mice (n = 9–10 in each group) were immunized twice 3 weeks apart, with 30 µg of NPm, NPwt, OVX836, or buffer (control) by the IM route. At D28, anti-CD45 Abs were administered by the IV route to label vascular cells. **(A)** Representative flow cytometry analysis of circulating (CD45+) and resident (CD45−) distribution in the lung after immunization. **(B)** Representative flow cytometry analysis of circulating and resident lung NP_366–374_-specific CD8+ T-cells using H2-Db NP_366–374_ pentamer staining. **(C)** Ratio resident/circulating of percent H-2Db NP_366–374_ Pentamer + CD8+ T-cells of two independent experiments. **(D)** Representative overlay plots of flow cytometric analysis showing distribution of lung-resident H-2Db NP_366–374_ Pentamer^+^CD8^+^CD45^−^ T-cell (red dots) TRM generated following vaccination among the CD69+CD103+ (black) population. **(E)** Graph representing percent resident H-2Db-NP_366–374_ Pentamer+ among CD8+CD69+CD103+ cells in the lung after vaccination of two pooled independent experiments. Individual data and mean ± SEM are represented. Differences were assessed by one-way ANOVA followed by Tukey’s multiple comparison test. *p < 0.05, **p < 0.01, ***p < 0.001, ****p < 0.0001.

Because the NP-specific CD8+ T-cells following vaccination was mainly persistent in the lung (**Figure 2A**), we investigated the presence of lung TRM CD8^+^ T-cells that could rapidly control viral infection upon challenge (18), as well as their cytotoxic function. As shown in **Figure 5A**, when using *in vivo* CD45 staining to distinguish circulating and tissue-resident cells (23), we observed that OVX836 induced more resident CD8+ T-cells compared to NPwt and NPm. We also observed a significantly higher percentage of resident H-2Db NP_366–374_ Pentamer^+^ among CD8^+^ T-cells following OVX836 immunization (**Figure 5B**). Likewise, the ratio of resident/circulating H-2Db NP_366–374_ Pentamer^+^ CD8^+^ T-cells percentages was significantly higher after OVX836 vaccination compared to NPwt and NPm (p <0.01 and 0.001, respectively) (**Figure 5C**). CD103 and CD69 integrins are markers of the TRM population in tissue (24). Representative flow cytometry analysis for the expression of CD103 and CD69 markers on NP_366–374_ Pentamer^+^ CD8^+^ T-cells is shown in **Figure 5D**. We found a higher abundance of H-2Db NP_366–374_ Pentamer^+^ among CD8^+^ CD69^+^CD103^+^ T-cells following vaccination with OVX836 compared to NPwt (p <0.05) and NPm (p <0.0001) vaccinated mice (**Figure 5E**). To our knowledge, this is the first demonstration that a NP protein vaccine favors the induction of TRM CD8^+^ T-cells.

Of note, the effector memory (TEM) (*CD44*high*CD62L*neg) populations significantly expanded after OVX836 immunization compared to NPwt and NPm (**Supplementary Figure S2B**, gating strategy in **Figure S2A**). TEM cells are cytotoxic and present in the circulation. They can be easily recruited to sites of inflammation and could rapidly control viral infection upon challenge (25).

**Figure 6:**
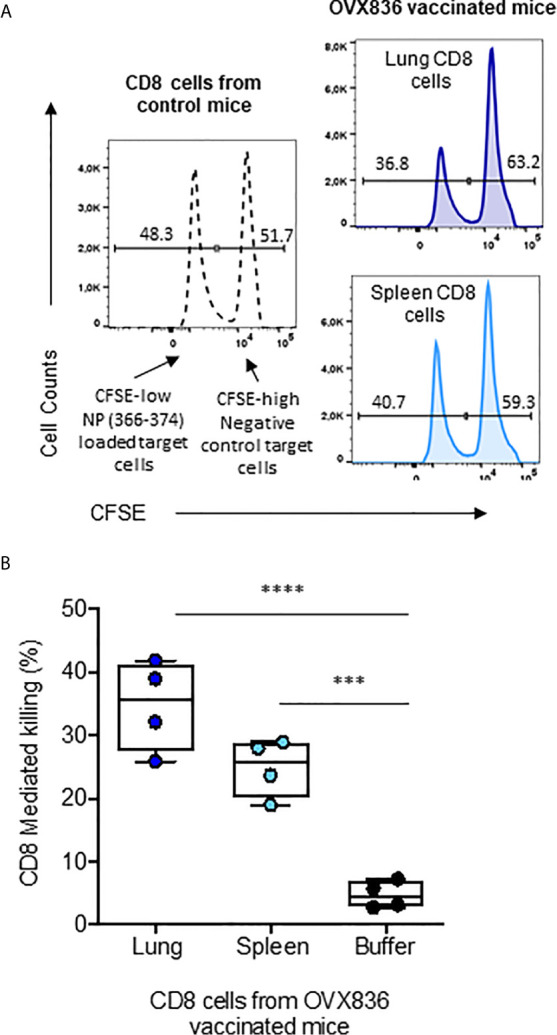
NP-specific CD8+ T-cells from OVX836-immunized mice are cytotoxic effector cells. **(A)** Splenocytes from naive C57/B6 mice were labelled with CFSE: the CFSE^low^ peak corresponds to NP_366–377_-pulsed target cells, the CFSE^high^ peak corresponds to unpulsed target cells. CFSE target cells were mixed at a 1:1 ratio and incubated for 16 h with sorted CD8+ T-cells isolated from the lungs and spleens of OVX836-vaccinated mice to measure antigen-specific CD8+ T-cell-mediated killing. **(B)** The data are representative of two separate experiments yielding similar results, represented in box-and-whisker plots (min and max, all points are shown) in the right panel. Differences were assessed by one-way ANOVA followed by Tukey’s multiple comparison test. Significance was set at ***p < 0.001, ****p < 0.0001.

We then assessed the cytotoxic potency of the CD8+ T-cells (**Figure 6A**). CFSE-labeled splenocytes loaded with NP_366–374_ (CFSE^high^) or negative controls (CFSE^low^) were used as target cells for effector CD8^+^ T-cells isolated from the lung and spleen of OVX836-immunized mice (**Figure 6A**). We found that CD8^+^ T-cells isolated from the lung and spleen of OVX836-immunized mice displayed significant cytotoxic function with about 35% (lung) and 25% (spleen) of killing activities (**Figure 6B**). Thus, the OVX836 vaccine induces NP-specific cytotoxic CD8^+^ T-cells in the lung and spleen.

### OVX836 Heptameric NP Vaccine Generates Lung Tissue-Resident Memory CD8+ T-Cells for Cross-Protection Against Influenza

**Figure 7:**
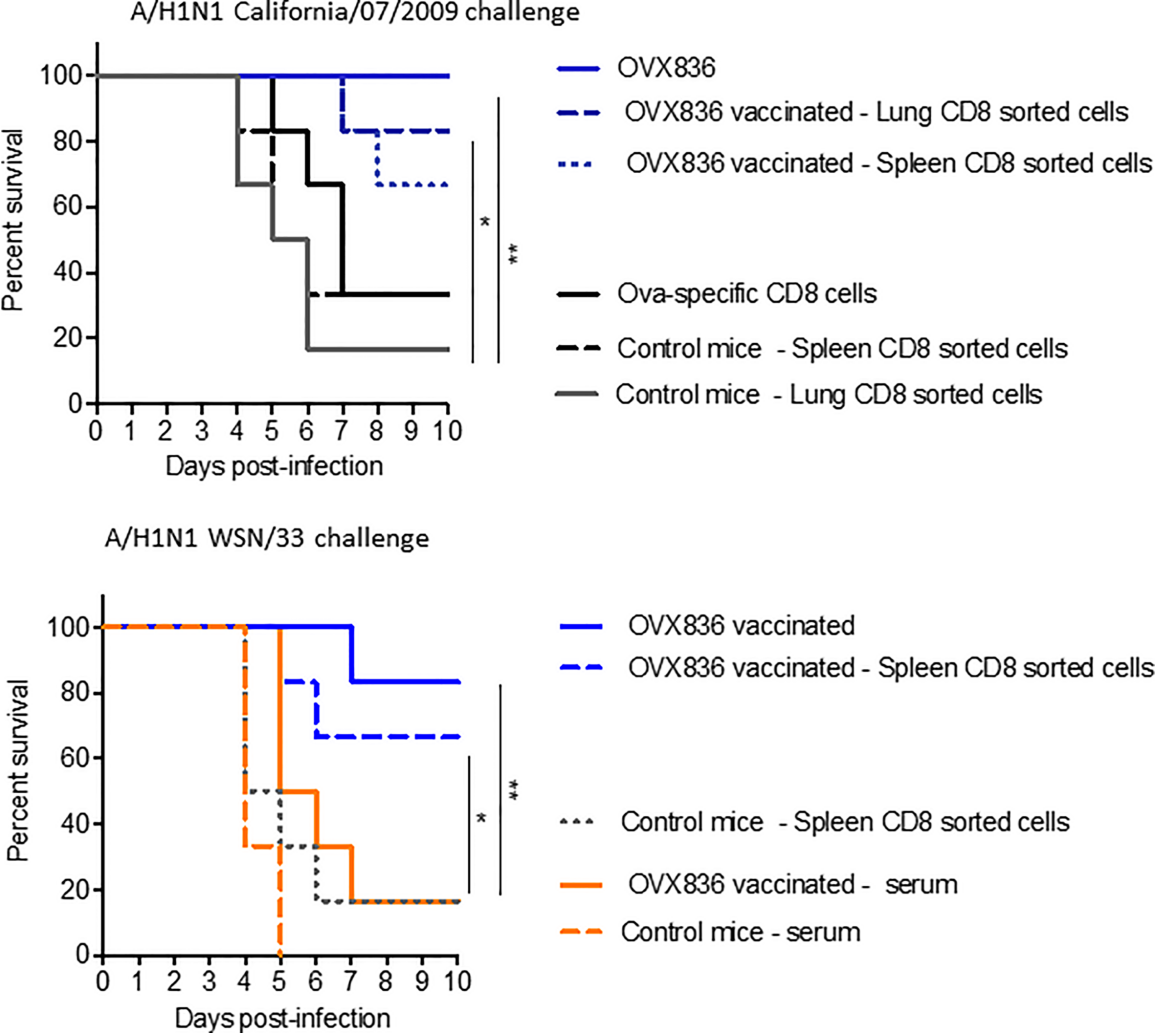
NP-specific CD8+ T-cells protect mice from lethal influenza challenges. **(A, B)** Donor C57BL/6 mice (n = 6–7 in each group) received two IM immunizations (D0, D21) with OVX836 (30 μg), buffer (control mice), or OVA (10 μg) + IFA. Spleens and/or lungs were collected from the donor mice at D28 to obtain CD8+ T-cells for the transfer **(A)**. Lungs to obtain CD8+ T-cells and serum were collected from the donor mice at D36 for the transfer **(B)**. Naive C57BL/6 recipient mice (n = six in each group) received 5 × 10^5^ lung-enriched CD8+ T-cells by the IV route or 300 μl of serum by the intraperitoneal (IP) route. Some 24 h after this adoptive transfer, all recipient mice were IN infected with 10^4.7^ TCID_50_/20 µl of the influenza viral strain H1N1 A/California/07/2009 **(A)** or H1N1 A/WSN/33 **(B)**. OVX836-vaccinated mice were used as positive controls in each experiment. Mice were then observed daily for clinical signs and body weight changes for 10 days. Percent survival rates are presented. *p < 0.05, **p < 0.01 by Log-Rank (Mantel–Cox) test.

In order to determine the role of the different immune arms in the protection against lethal influenza challenge following OVX836 vaccination, we performed adoptive transfer of CD8+, CD4+ T-cells, or serum from vaccinated mice into recipient mice prior to influenza challenge. The experimental scheme is presented in **Supplementary Figure 3A**. Protection against the lethal A/California/07/2009 (H1N1) challenge was observed following adoptive transfer of sorted CD8+ T-cells from either lungs or spleens of OVX836-vaccinated mice, and not after transfer of either CD8+ T-cells from OVA-immunized mice or from unvaccinated mice (**Figure 7A**). Protection against the lethal A/WSN/1933 (H1N1) challenge was confirmed following adoptive transfer of sorted CD8+ T-cells from the spleens of OVX836-vaccinated mice (**Figure 7B**). In this lethal influenza challenge, the transfer of serum (**Figure 7B**) or sorted CD4+T cells from OVX836-immunized mice into recipient mice did not confer significant protection (**Supplementary Figure 3B**). In accordance with our previous findings, we observe cross-protection, since mice vaccinated with OVX836 or receiving CD8+ T-cells from OVX836 (containing NP of A/WSN/1933(H1N1) were protected after both Influenza A/WSN/1933(H1N1) virus (**Figure 7B**) and Influenza A/California/07/2009 (**Figure 7A**) challenges.

## Discussion

We have demonstrated that OVX836, a heptameric form of the influenza NP protein, has a high potency for the induction of both peripheral and lung-associated resident CD8+ T-cells as assessed by IFNγ production and cytotoxic function. The persistence of protective CD8+ T-cells is a striking feature of OVX836 compared to monomeric and trimeric NP. The identification of the mechanism of protection conferred by OVX836 provides a paradigm of the importance of NP CD8^+^ T-cell-mediated vaccine for heterosubtypic protection (5, 19).

Some vaccination strategies attempt to promote induction of CD8^+^ T-cell responses either by targeting the viral proteins that promote a cell-mediated response (M1, NP) or using vaccines in the form of particles (virosomes, virus-like particles, viral vectors, DNA, live attenuated influenza vaccine) to simultaneously promote humoral and cellular responses (10). In addition, the use of alternative routes of administration of vaccine formulations has provided evidence for the induction of systemic CD8+ T-cell responses (12, 26). Induction of lung-tissue CD8+ T-cell responses using protein-based vaccines remains challenging for vaccinologists. Inactivated influenza vaccines and LAIV are the two approved classes of influenza vaccine administered by the IM and IN routes, respectively (27). Both vaccines generate HA-specific antibodies while T-cell responses are significantly higher with LAIV (28). A single immunization with LAIV induces TRM, while vaccination with an inactivated influenza vaccine by a systemic or IN route is not sufficient to induce such T-cell responses in the lung (18). One of the main issues discussed for LAIV vaccination in adults, however, is the presence of pre-existing Influenza-specific memory responses in the lung that may affect vaccine efficacy by the IN route. Our finding demonstrates that conventional IM administration of OVX836 can induce memory CD8^+^ T-cells, both at the systemic level and in lung tissue, as well as protective efficacy against heterosubtypic influenza viruses.

The role of TRM in the lung has been evaluated for long-term protection in murine models, a question that cannot currently be addressed in humans (18, 29). Lessons learned from murine models of influenza infection have shown that the anti-viral activity of CD8^+^ T-cells is strongly dependent on their ability to migrate and localize in the lung while the expansion is detected in the secondary lymphoid tissue (30). After OVX836 vaccination, we found that CD8^+^ T-cells isolated from spleen and lung tissue display cytotoxic functions and IFNγ production. These cells can protect the animal against lethal influenza challenges, suggesting peripheral expansion of these cells into the secondary lymphoid tissue. Adoptive transfer experiments showed that lung-associated CD8^+^ T-cells following OVX836 vaccination can protect the animals, whereas serum and CD4^+^ T-cells from immunized mice were not similarly protective in the influenza challenge model. It has been shown that multiple mechanisms of effector CD8^+^ T-cells can contribute to protection, including the release of anti-viral cytokines and perforin/granzyme, as well as the activation of the Fas/FasL pathway (31, 32). Despite the fact that adoptive transfer of CD4^+^ T-cells isolated from OVX836-immunized mice did not confer full protection against influenza infection in our vaccination model, it has been proposed that CD4^+^ T-cells guide the formation of TRM in the lung during influenza infection (33).

Antigen-specific CD8+ T-cells can be divided into central, effector, and resident memory cells based on the expression of CD62L, CCR7, CD69, and CD103. TRM expressing CD103+CD69+ are a highly specialized TRM population and are involved in viral clearance both in humans and in mice (34, 35). We demonstrated that OVX836 vaccination is more efficient than monomeric mutant NP (NPm) and trimeric wild-type NP (NPwt) at inducing CD8^+^ TRM in the lung tissue. However, the mechanism for induction of TRM is currently unknown, as is the reason why these CD8^+^ T-cells are localized in the lung. The site of antigen encounter as well as its transport and presentation to T-cells might play a role in programming T-cell migration to the tissue (26, 36). One could hypothesize that heptamerization would promote better uptake and processing for MHC-class I presentation on APC, leading to a higher number of antigen-specific CD8^+^ T-cells. According to this hypothesis, we found that OVX836-loaded DC induced a higher effector CD8^+^ T-cell activation compared to NPm and NPwt, suggesting involvement of DC cells in favoring CD8+ T-cell responses following OVX836 immunization. Of note, NPm, NPwt, and OVX836 exhibit different physicochemical characteristics. NPm is monomeric due to the mutation of two amino acids in the NP sequence (E339A/R416A), as the ionic bond between R416 and E339 involved in NP oligomerization is then disrupted (19, 21). This results in particle sizes smaller than 7 nm and greater sensitivity to thermal denaturation. NPwt is trimeric, with an average particle diameter smaller than 15 nm, and more stable to thermal denaturation. OVX836 forms heptamers due to the OVX313 moiety and higher-order structures related to the self-associative properties of NP, thus exhibiting the largest particle size (e.g. 20–40 nm) among the three NP proteins, without detrimental effects to its stability. In addition, ion exchange chromatography shows that OVX836 (apparent p*I* 9.8) is more cationic than NPwt (apparent p*I* 9.5), mainly due to the presence of arginine and lysine residues in OVX313. NPm is overall less cationic, with probably less cationic groups on its surface (apparent p*I* 8.6, data not shown). Thus, the properties of OVX836—higher particle size, high thermal stability, and more surface cationic groups—might contribute to DC maturation and activation during immunization. Further studies need to be performed on the mechanism of antigen-presentation by conventional DC for cross-presentation to T-cells.

The persistence and durability of memory responses is the cornerstone of successful vaccination. The presence of memory CD8^+^ T-cells is potentially one of the contributing reasons that most healthy unvaccinated adults do not experience severe influenza disease on more than a few occasions. These T-cells might provide rapid and highly effective protective immunity during re-encounter of pathogens (15). In addition, CD8^+^ T-cells can recognize more conserved epitopes of pathogens and provide protection against several viral proteins (37, 38). CD8^+^ T-cell responses might diminish the morbidity and mortality typically caused by a newly emerging viral subtype (39, 40). In humans, resident effector cytotoxic T-cells are generated following multiple influenza infections. Epitopes of NP that are recognized by human cytotoxic T-cells have been identified (41). In addition, diverse TCR profiles of TRM cells and a high degree of clonal sharing with other CD8+ T-cell populations with polyfunctionality have been found in human lung tissue (42). This characteristic might be important for protection against the generation of viral-escape mutants. In pandemic situations or when influenza drifted strains circulate, available vaccines against conserved proteins of influenza virus would help to protect the population. Vaccines that promote cytotoxic T-cell responses in the lung could prevent seasonal or pandemic influenza, as a stand-alone and in combination with antibody approaches. Although cellular immunity is not sterilizing, it could contribute to significantly decreasing influenza illness, hospitalization, and death in humans (41). OVX836 is currently under clinical development: phase I was completed and Phase IIa was recently finalized (ClinicalTrials.gov Identifier: NCT03594890, NCT04192500).

## Data Availability Statement

The datasets presented in this study can be found in online repositories. The names of the repository/repositories and accession number(s) can be found in the article/**Supplementary Material**.

## Ethics Statement

The animal study was reviewed and approved by CECCAPP_ENS_2018_019, Lyon, France.

## Author Contributions

Study conceptualization and supervision (JC, BC, FN, AV, DG, and FH). Methodology and experimental design (JC, BC, JB, SD, YL, JM, and FN). Conduction of experiments (JC, JB, MH, MC, CR, and AI). Collection and analysis of data (JC, BC, and JB. Manuscript writing (JC, BC, JB, and FN). All authors contributed to the article and approved the submitted version.

## Funding

This project has received funding from Bpifrance (grant nos. DOS0058200/00, DOS0080075/00, & DOS0080076/00), from the European Union’s Horizon 2020 research and innovation program under grant agreement no. 961112 and from the Region Auvergne-Rhône-Alpes. Bpifrance was not involved in the study design, collection, analysis, interpretation of data, the writing of this article or the decision to submit it for publication.

## Conflict of Interest

AV, DG, and FN are employed by and shareholders of Osivax. JC and FH are employed by Osivax and inventors of some of Osivax’ patents. JC, JB, MC, CR, MH, and AI are employed by Osivax. BC is member of the advisory board of Osivax and receives honoraria.

The remaining authors declare that the research was conducted in the absence of any commercial or financial relationships that could be construed as a potential conflict of interest.
