# Supplementary figures and images for: OVX836 Heptameric Nucleoprotein Vaccine Generates Lung Tissue-Resident Memory CD8+ T-Cells for Cross-Protection Against Influenza
Source: Front Immunol. 2021 Jun 10;12:678483. doi: 10.3389/fimmu.2021.678483 (PMC8223747; doi:10.3389/fimmu.2021.678483)

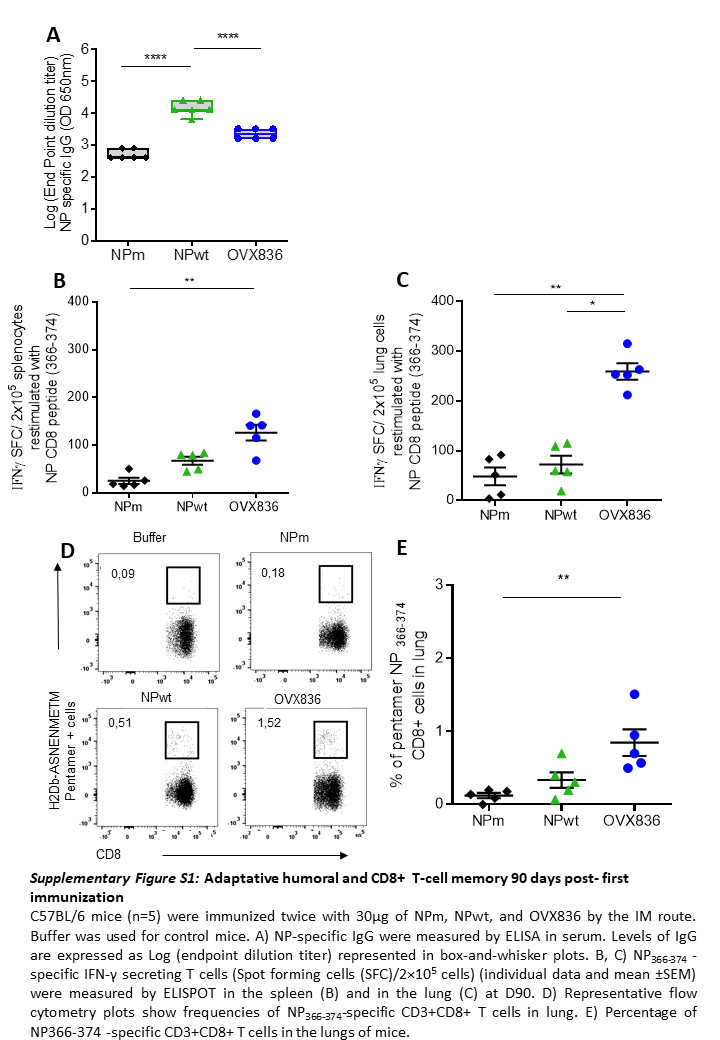

Supplement: Supplementary file 1 [file Image_1.tif]

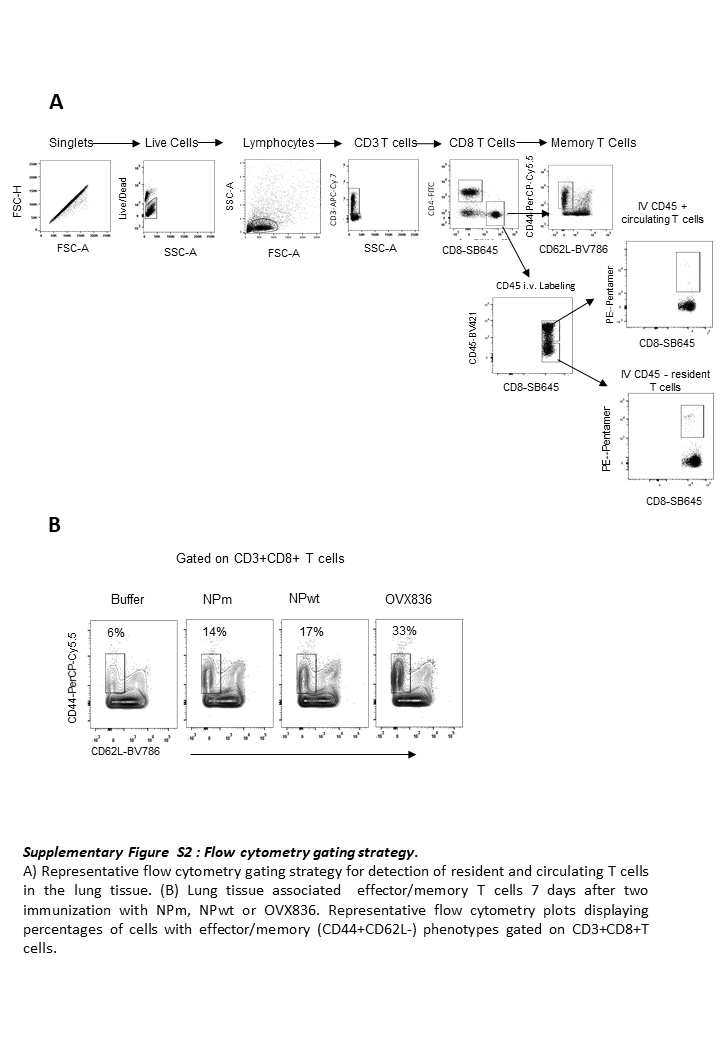

Supplement: Supplementary file 2 [file Image_2.tif]

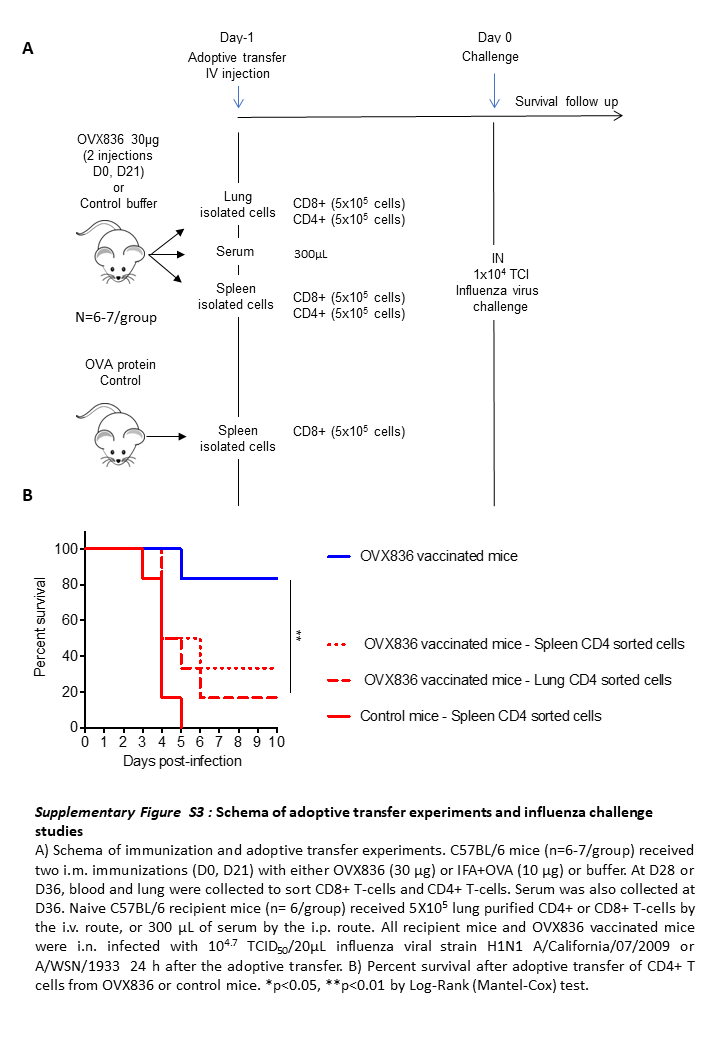

Supplement: Supplementary file 3 [file Image_3.tif]
